# Supplementary material for: Inhibition of Digestive Enzymes and Antioxidant Activity of Extracts from Fruits of Cornus alba, Cornus sanguinea subsp. hungarica and Cornus florida–A Comparative Study
Source: Plants (Basel). 2020 Jan 18;9(1):122. doi: 10.3390/plants9010122 (PMC7020211; doi:10.3390/plants9010122)
Supplement: Supplementary file 1 [file plants-09-00122-s001.pdf]

Article

Inhibition of digestive enzymes and antioxidant activity of extracts from fruits of *Cornus alba*, *Cornus sanguinea* subsp. *hungarica* and *Cornus florida* – a comparative study

Joanna Truba[1], Iwona Stanisławska[2], Marta Walasek[1], Wioleta Wieczorkowska[1], Konrad Woliński[3], Tina Buchholz[4], Matthias F. Melzig[4], Monika E. Czerwińska[5,*]

[1] Student Scientific Association, Department of Pharmacognosy and Molecular Basis of Phytotherapy, Medical University of Warsaw, 1 Banacha street, 02-097 Warsaw, Poland
[2] Department of Bromatology, Medical University of Warsaw, 1 Banacha street, 02-097 Warsaw, Poland
[3] **Polish Academy of Sciences Botanical Garden, Center for Biological Diversity Conservation in Powsin,** 2 Prawdziwka street, 02-973 Warsaw, Poland
[4] Institute of Pharmacy, Freie Universitaet Berlin, 2+4 Koenigin-Luise street, D-14195 Berlin, Germany
[5] Department of Pharmacognosy and Molecular Basis of Phytotherapy, Medical University of Warsaw, 1 Banacha street, 02-097 Warsaw, Poland

*Corresponding author:
Monika E. Czerwińska, Department of Pharmacognosy and Molecular Basis of Phytotherapy, Medical University of Warsaw, 1 Banacha street, 02-097 Warsaw, Poland
e-mail address: mczerwinska@wum.edu.pl

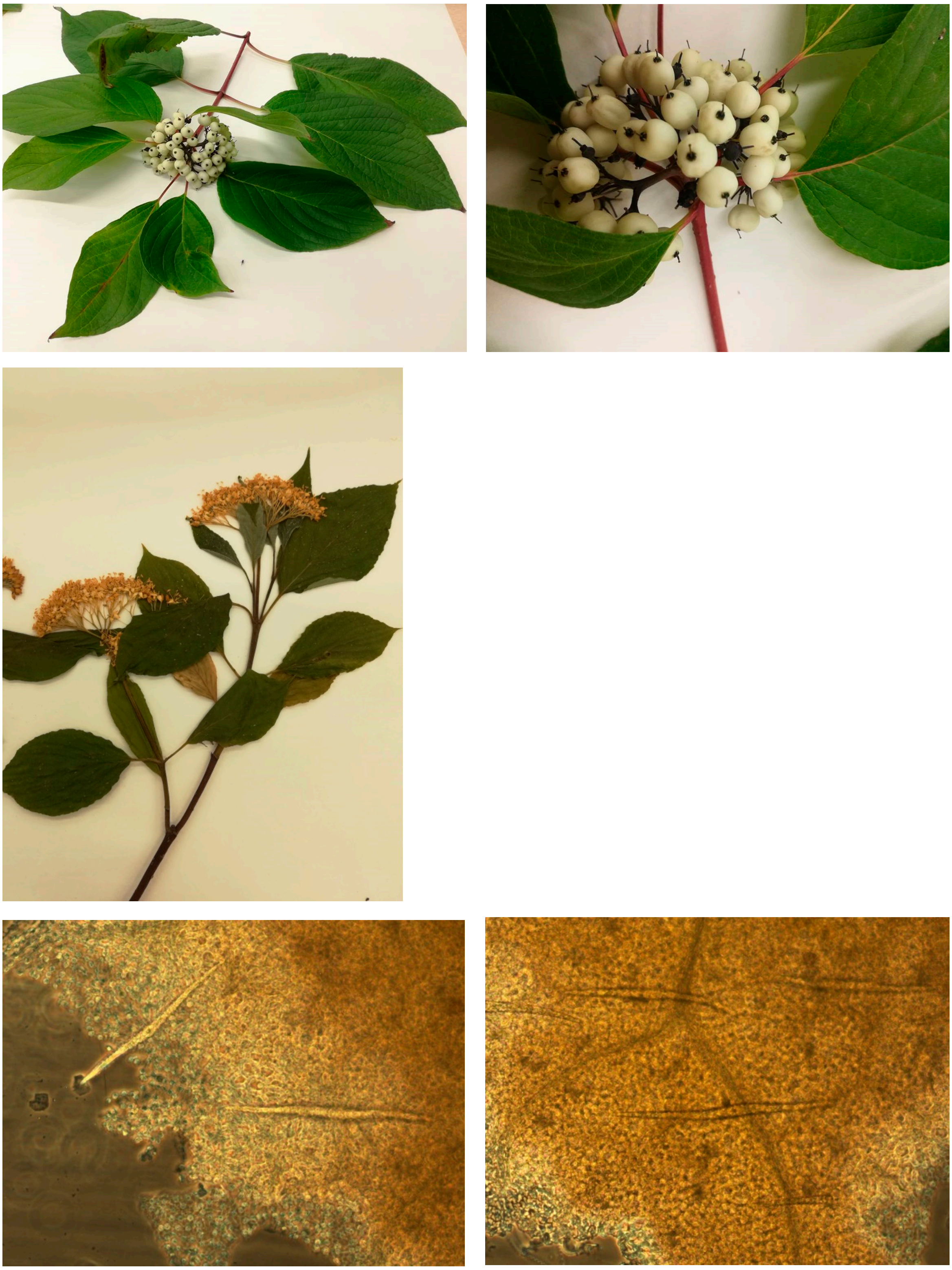

Figure S1. Morphological and anatomical characteristics of *Cornus alba* L. plant materials (leaves) in the optical microscope.

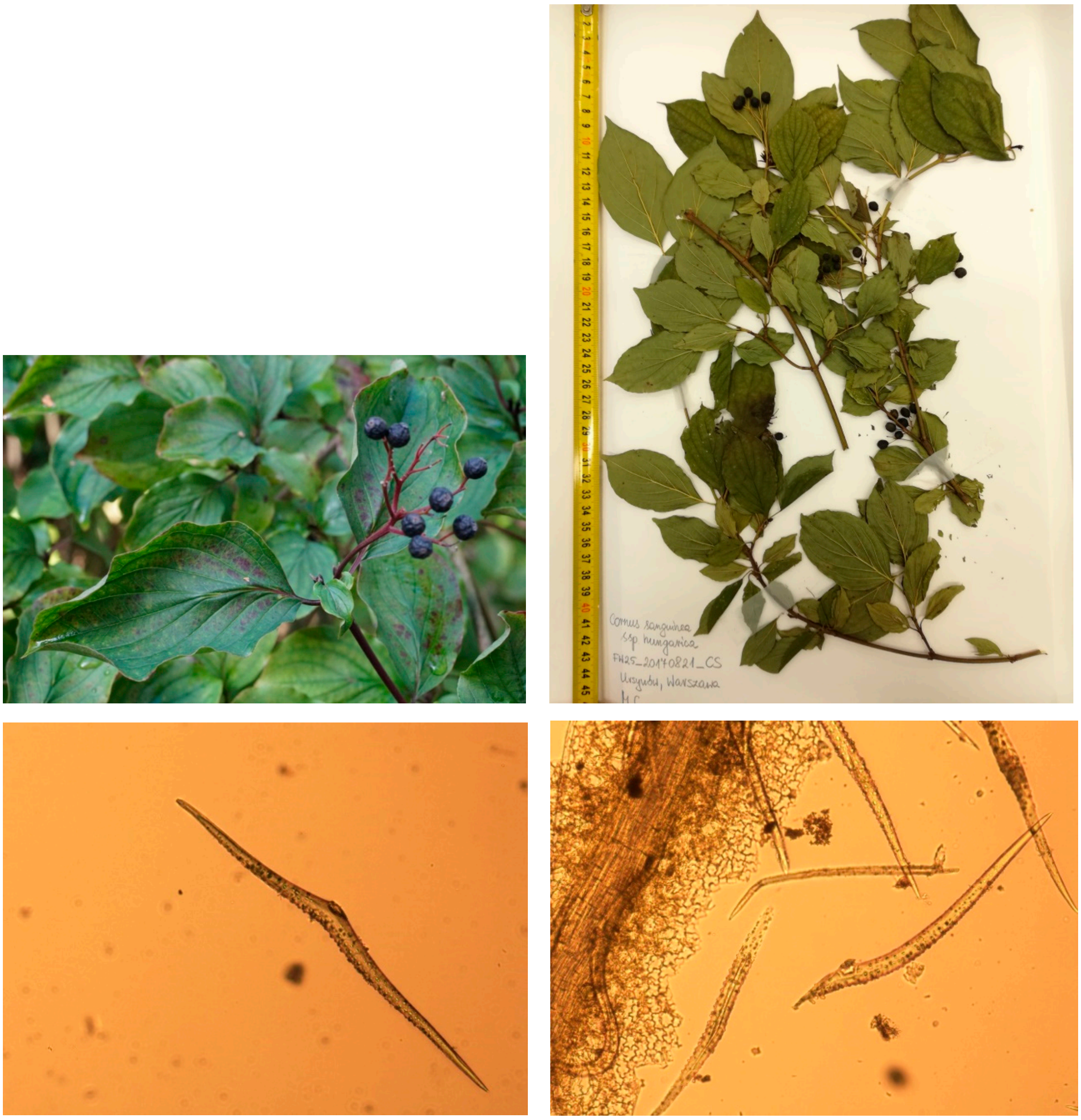

Figure S2. Morphological and anatomical characteristics of *Cornus sanguinea* L. subsp. *hungarica* plant materials (leaves) in the optical microscope.

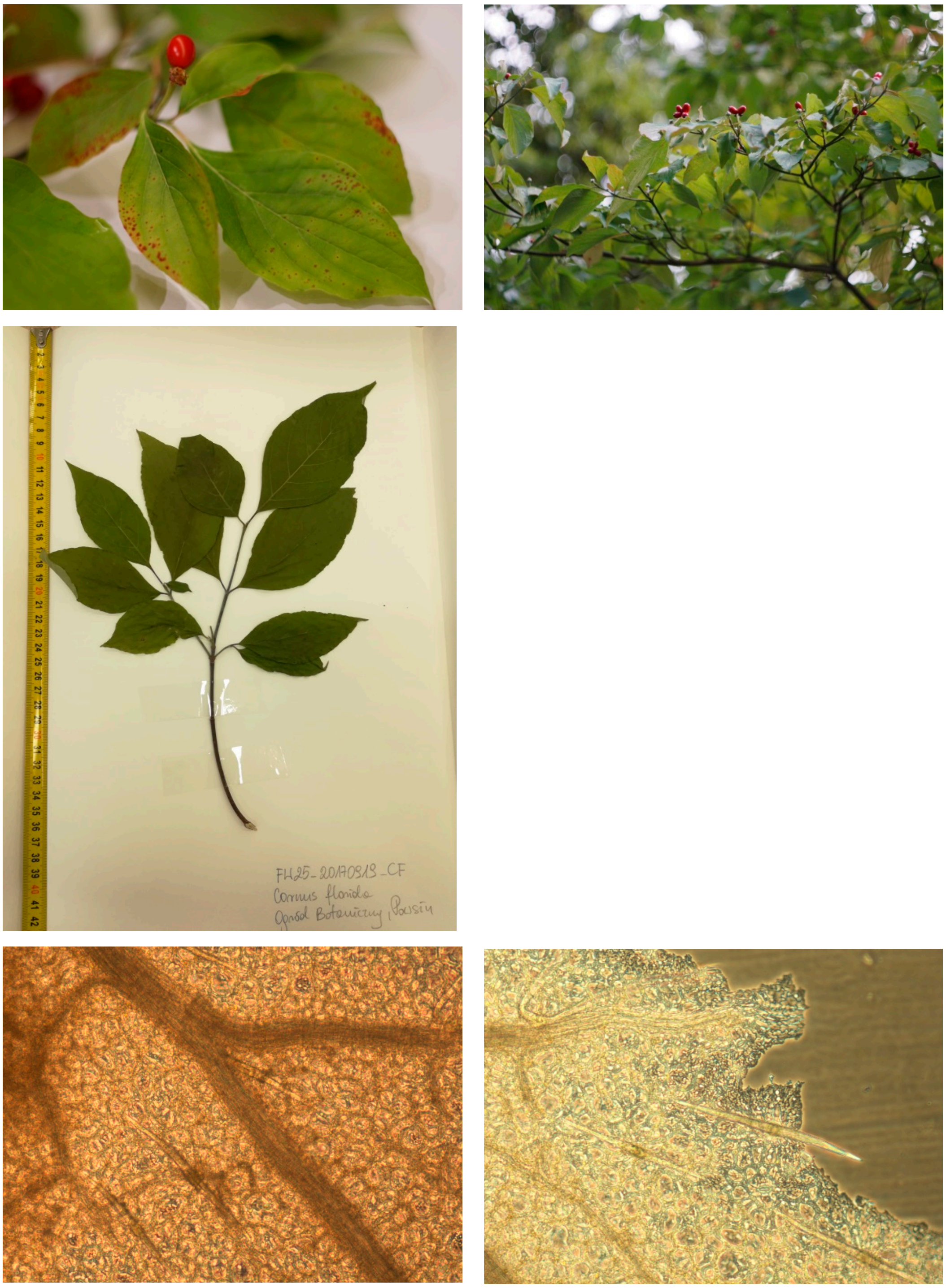

Figure S3. Morphological and anatomical characteristics of *Cornus florida* L. plant materials (leaves) in the optical microscope.

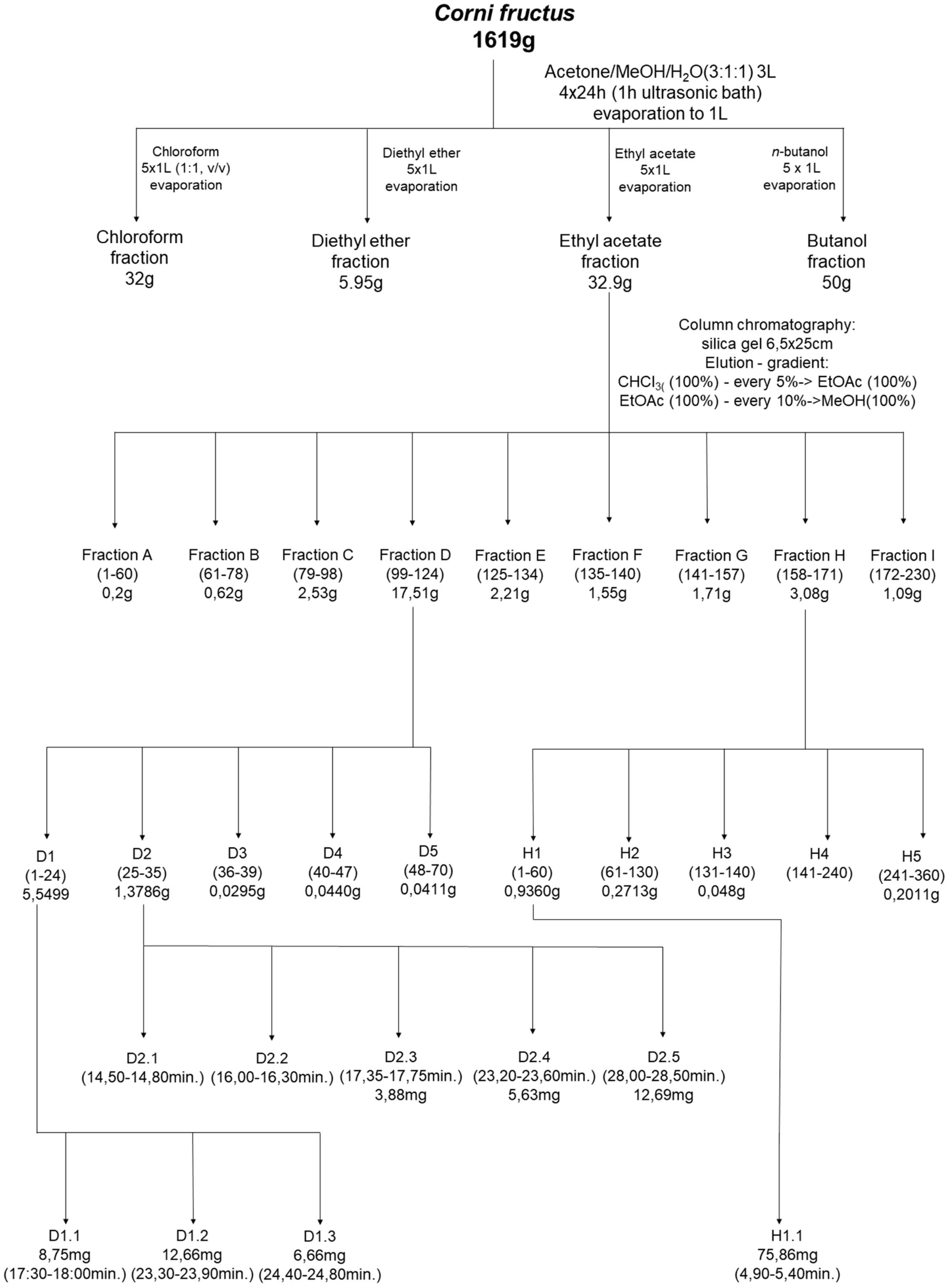

Figure S4. The draft of isolation of compounds from acetone-methanol-aqueous (3:1:1, v/v/v) extract from fruit of *Cornus alba*.

NMR spectra of isolated compounds

1. **Compound D2_1 (chlorogenic acid)**

The compound D2_1 was identified as 5-*O*-coumaroylquinic acid. Its structure was confirmed based on the data available in the literature [1].

2. **Compound D2_3 (5-*O*-coumaroylquinic acid)**

^{1}H NMR (300 MHz, CD_3OD) δ 7.63 (d, *J* = 15.8 Hz, H-7'), 7.46 (d, *J* = 8.5 Hz, H-2',6'), 6.80 (d, *J* = 8.5 Hz, H-3',5'), 6.34 (d, *J* = 16.0 Hz, H-8'), 5.36 (s, H-5), 4.16 (s, H-3), 3.73 (dd, *J* = 3.6, 8.7 Hz, H-4), 2.16 (m, H-2), 2.10 (m, H-6).
The compound D2_3 was identified as 5-*O*-coumaroylquinic acid. Its structure was confirmed based on the data available in the literature [2].

3. **Compound D2_4 (5-*O*-(E)-*p*-coumaroylquinic acid methyl ester)**

^{1}H NMR (300 MHz, CD_3OD) δ 7.60 (d, *J* = 16.0 Hz, H-7'), 7.46 (d, *J* = 8.6 Hz, H-2', H-6'), 6.81 (d, *J* = 8.6 Hz, H-3', H-5'), 6.29 (d, *J* = 16.0 Hz, H-8'), 5.29 (ddd, *J* = 7.7, 7.7, 4.5 Hz, H-5), 4.14 (dt, *J* = 6.6, 3.4 Hz, H-3), 3.73 (dd, *J* = 7.6, 3.2 Hz, H-4), 2.19 (m, H_2-6), 2.02 (dd, *J* = 13.7, 6.7 Hz, H_2-6), 1.30 (s, OCH_3).

The compound D2_4 was identified as 5-*O*-(E)-*p*-coumaroylquinic acid methyl ester. Its structure was confirmed based on the data available in the literature [3].

4. **Compound D2_5 (kaempferol 3-*O*-glucuronide 6''-methyl ester)**

^{1}H NMR (300 MHz, CD_3OD) δ 8.01 (d, *J* = 8.9 Hz, H-2' e H-6'), 6.87 (d, *J* = 8.9 Hz, H-3' e H-5'), 6.40 (d, *J* = 1.9 Hz, H-8), 6.20 (d, *J* = 1.9 Hz, H-6), 5.23 (d, *J* = 7.3 Hz, H-1''), 3.65 (s, OCH_3), 3.60 – 3.34 (overlapping uronic acid moiety).
The compound D2_5 was identified as kaempferol 3-*O*-glucuronide-6''-methyl-ester. Its structure was confirmed based on the data available in the literature [4].

5. **Compound H1_1 (hydroxytyrosol glucoside)**

^{1}H NMR (300 MHz, CD_3OD) δ 6.62 (d, *J* = 3.1 Hz, H-2), 6.58 (s, H-5), 6.49 (d, *J* = 2.9 Hz, H-6), 4.31 (d, *J* = 7.7 Hz, H-1'), 4.05 (m, H-8a), 3.88 (d, *J* = 1.9 Hz, H-6'a), 3.79 – 3.65 (overlapping H-5', H-6'b, H-8b), 3.36 – 3.28 (overlapping H-2', H-3'), 3.19 (t, *J* = 8.3 Hz, H-4'), 2.87 (t, *J* = 7.3 Hz, H-7).
The compound H1_1 was identified as hydroxytyrosol glucoside. Its structure was confirmed based on the data available in the literature [5].

References

1. Nakamura, K.; Ogasawara, Y.; Endou, K.; Fujimori, S.; Koyama, M.; Akano, H. Phenolic compounds responsible for the superoxide dismutase-like activity in high-brix apple vinegar. *J. Agric. Food Chem.* **2010**, *58*, 10124-10132.
2. Lu Y., Sun Y., Foo L.Y. et al. 2000. Phenolic glycosides of forage legume *Onobrychis viciifolia*. *Phytochemistry* 55, 67-75.
3. Nakamura S., Fujimoto K., Matsumoto T., et al. 2013. Acylated sucroses and acylated quinic acids analogs from the flower buds of *Prunus mume* and their inhibitory effect on melanogenesis. *Phytochemistry*. 92, 128-36.
4. Pacifico S., D'Abrosca B., Scognamiglio M., et al. 2013. Antioxidant polyphenolic constituents of *Vitis*× *Labruscana* cv.'Isabella'leaves. *Open Nat Prod J.* 6, 5-11.
5. Romero C., Brenes M., García P., et al. 2002. Hydroxytyrosol 4-*β*-D-glucoside, an important phenolic compound in olive fruits and derived products. *J Agric Food Chem.* 50, 3835-9.
